# Preparation and Characterization of Microemulsions Based on Antarctic Krill Oil

**DOI:** 10.3390/md18100492

**Published:** 2020-09-25

**Authors:** Jiawen Zhao, Kening Jiang, Yixuan Chen, Juan Chen, Yangfan Zheng, Huilin Yu, Jiajin Zhu

**Affiliations:** Institute of Food Science, College of Biosystems Engineering and Food Science, Zhejiang University, Hangzhou 310058, Zhejiang, China; 21813084@zju.edu.cn (J.Z.); 3140101878@zju.edu.cn (K.J.); 11713038@zju.edu.cn (Y.C.); 11613037@zju.edu.cn (J.C.); 21713068@zju.edu.cn (Y.Z.); yuhl0323@126.com (H.Y.)

**Keywords:** microemulsion, Antarctic krill oil, formula selection, characterization

## Abstract

Antarctic krill oil is high in nutritional value and has biological functions like anti-inflammation and hypolipidemic effects. But it has and unpleasant smell, and unsaturated fatty acids are prone to oxidative deterioration. Its high viscosity and low solubility in water make it difficult for processing. Microemulsion can be a new promising route for development of krill oil product. We determined a formula of krill oil-in-water microemulsion with krill oil: isopropyl myristate = 1:3 as oil phase, Tween 80:Span 80 = 8:2 as surfactant, ethanol as co-surfactant and the mass ratio of surfactant to co-surfactant of 3:1. After screening the formula, we researched several characteristics of the prepared oil-in-water microemulsion, including electrical conductivity, microstructure by transmission electron microscope and cryogenic transmission electron microscope, droplet size analysis, rheological properties, thermal behavior by differential scanning calorimeter and stability against pH, salinity, and storage time.

## 1. Introduction

Krill oil is extracted from a species of Antarctic krill, *Euphausia superba*, which is mainly active in the deep ocean and can be a food source for several larger marine animals (e.g., seals and whales) [1]. Krill oil (KO) is rich in omega-3 polyunsaturated fatty acid (PUFA) and has security as an unconventional food source for ω-3 PUFAs [2,3], generally recognized as safe (GRAS) by the American Food and Drug Administration (FDA) [4]. As the main component of the cell membrane is primarily phospholipids (PL), as compared to fish oil (FO), the long-chain ω-3 PUFAs in krill oil, such as eicosapentaenoic acid (EPA) and docosahexaenoic acid (DHA), are more easily absorbed and utilized by the human body due to the combination with PL, while DHA and EPA in FO mainly exist with the combination of triacylglycerol (TAG) [2,5,6]. In the respect of hormone level, the hormonal fluctuation caused in polycystic ovary syndrome (PCOS) was brought to nearly normal levels by omega-3 fatty acid present in the krill oil, thus showing its potential effect and an alternative to metformin in treating PCOS [7]. Ferhatoğlu reported that supplementing patients with krill oil before colorectal surgery may reduce some risk factors for anastomotic leakage, accelerating wound healing and reducing excessive inflammation [8]. There are also considerable amounts of bioactive components in krill oil, such as astaxanthin, sterols, vitamin A, tocopherols, flavonoids, and minerals [9,10]. Many studies have suggested the health benefits of Antarctic krill oil. Zhan found that supplementing Antarctic krill oil (AKO) in the diet for 21 days was beneficial to the longitudinal bone growth of adolescent male mice by the growth hormone-insulin-like growth factor-1 (GH-IGF-1) pathway, which indicates that AKO might be a potential functional food and could improve adolescents’ growth potential [11]. Yun used the maze to judge the learning and memory ability of rats, showing that the rats taking krill oil had superior performance to fish oil in the maze [12]. Krill oil has a protective function on cognitive function, and its active prevention of neurodegenerative diseases such as Alzheimer disease has attracted more and more attention [13,14,15]. Konagai also verified this finding in humans [14]. The results showed that subjects taking krill oil improved significantly the level of oxyhemoglobin in the memory test than the control group, suggesting that krill oil can delay brain degeneration by activating the dorsolateral prefrontal cortex of the brain. In terms of inhibiting apoptosis, Xiong found that krill oil can reduce oxidative damage and methamphetamine (METH)-triggered apoptosis, thereby attenuating the neurotoxicity induced by METH [16]. What is more, krill oil has been found to play an important role in lowering blood fat and lowering blood sugar [17,18,19,20,21,22]. The anti-inflammatory properties of krill oil have been proven over the years [23,24]. Bonaterra found that krill oil can reduce or even eliminate the lipopolysaccharide (LPS) binding ability and reduce the release of tumor necrosis factor -α (TNF-α) [25]. Other studies [19,25] also confirmed that krill oil had the ability to reduce C-reactive protein levels, which are the markers of systemic inflammation. Oxidative stress and inflammation are the main cause of knee osteoarthritis (OA) pathogenesis and krill oil could not only ameliorate knee pain [26] but also reduce systemic inflammation of the body caused by OA [27]. In terms of inflammatory bowel disease (IBD), ω-3 PUFA and astaxanthin in KO also show the significantly anti-inflammatory property in the management with promising results [28]. In addition, DHA in krill oil is an indispensable material for constructing and repairing retinal nerve cells [29,30]. Astaxanthin contained in krill oil also has an effect of improving eye function [31]. In the meanwhile, AKO could enhance the bioavailability of astaxanthin and the research on AKO would accelerate developing functional foods and special medical foods based on such scientific basis and theoretical support [32]. The antioxidant components of krill oil help the body protect the retina and relieve damage to photoreceptor cells. Due to its various benefits for body health, krill oil has a promising potential in applications for food [2,33], nutraceuticals, and pharmaceuticals as a novel food ingredient. In recent years, relevant krill oil products are very often seen in the forms of capsules, soft gels, tablets, and gummies as dietary supplements in the market [9].

However, Antarctic krill oil has an unpleasant smell, which reduces its acceptability. The unsaturated fatty acids and other components are prone to oxidation and deterioration [34,35,36]. The high viscosity and low water solubility of krill oil make the processing of krill oil difficult. So far, the research on Antarctic krill oil includes physiological functions, extraction technologies (including solvent type, nonsolvent type, super/subcritical fluid type, and enzyme-assisted type) [9], soft capsule formulation, and preparation of microcapsules by complex coacervation method [37,38], acute pore method, spray drying method [39], etc.

Microemulsion is a clear, transparent, or translucent and thermodynamically stable liquid-liquid dispersion system, highly dispersed and low in viscosity, and generally composed of water phase, oil phase, surfactant, and co-surfactant [40,41]. The formation of microemulsions is a spontaneous process [42,43]. As long as the composition of four phases is appropriate, a uniform transparent or slightly opalescent liquid can be formed just by slightly stirring [40]. In recent years, microemulsions have been used as a carrier of functional ingredients in the food industry [44] and potential drug carriers for oral, topical, and parenteral administration [45]. Using microemulsification technology to prepare stable Antarctic krill oil microemulsion, it can not only overcome many problems in processing restrictions, but also cover the shortcomings of the oil’s own stinky odor. The prepared KO microemulsion is low in toxicity, high in safety, and can be mass produced without special equipment. Therefore, the preparation and characterization of Antarctic krill oil microemulsion can provide a new way for the development of Antarctic krill oil products, which not only ensures the quality of krill oil products regarding appearance, stability, and nutritional value but also brings convenience for the actual product processing [46]. In addition, the droplet size of the microemulsion is below 100 nm, which is more conducive to human absorption. It is worth noting that due to the beneficial health effects of ω-3 PUFAs, KO is competitive as functional food ingredients and drug nanocarriers. Transport and delivery of various nutrients and bioactive food additives throughout the body might be facilitated, attributed to these structures [4].

This study aimed to explore the preparation of oil-in-water (O/W) Antarctic krill oil microemulsion and its characteristics. Appropriate oil phase, surfactant, and co-surfactant were screened by comparing the size of the microemulsion area in a pseudo-ternary phase diagram. After obtaining the best formula, the electrical conductivity was measured, the microstructure was observed by transmission electron microscopy, and droplet size analysis was completed. Rheological properties were studied by rotary rheometer, and thermal behavior was analyzed by differential scanning calorimeter. The stability of the O/W Antarctic krill oil microemulsion was investigated by analyzing the impact of salinity, pH, storage temperature, and time.

## 2. Results and Discussion

### 2.1. Determination of Antarctic Krill Oil O/W Microemulsion

**Figure 1:**
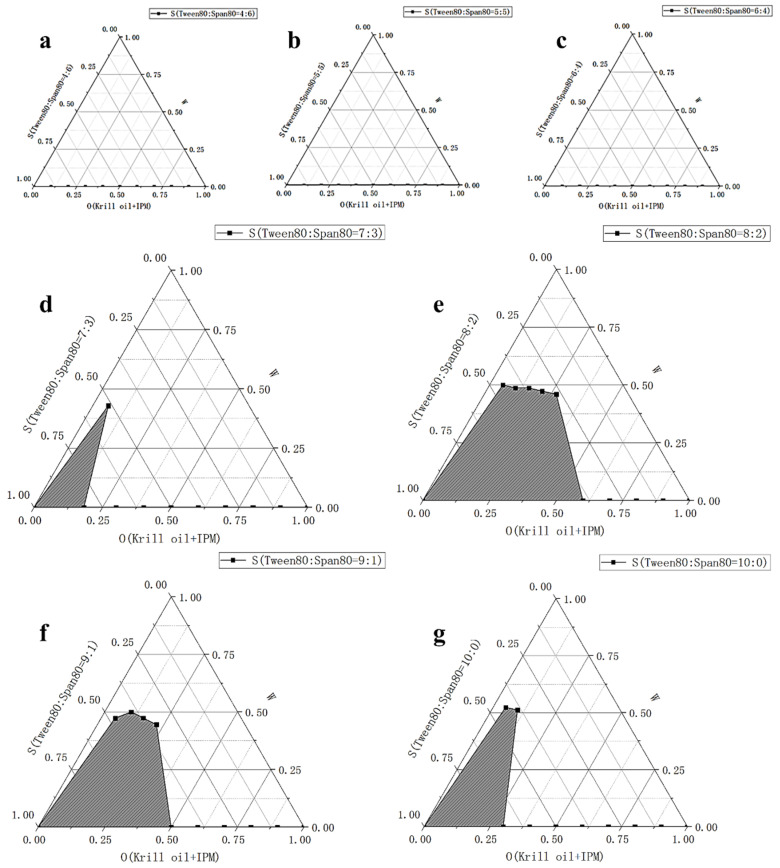
Microemulsion area in phase diagram for each ratio of surfactants (Tween80:Span80). (**a**) Tween80:Span80 = 4:6 (**b**) Tween80:Span80 = 5:5 (**c**) Tween80:Span80 = 6:4 (**d**) Tween80:Span80 = 7:3 (**e**) Tween80:Span80 = 8:2 (**f**) Tween80:Span80 = 9:1 (**g**) Tween80:Span80 = 10:0. S refers to the content of surfactants in the system; O refers to the content of il phase in the system; W refers to the content of water in the system. There was no microemulsion formed in a, b, or c. All microemulsions were formed in 25 ± 1 °C, unless otherwise specified.

**Figure 2:**
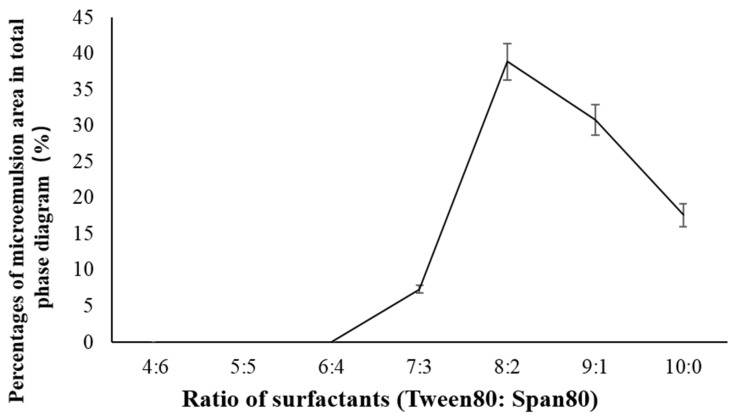
Percentages of microemulsion area in total phase diagram for each ratio of surfactants (Tween 80:Span 80).

We did three parts of experiments to select the best formula of krill oil microemulsion, including selection of surfactant, co-surfactant, and oil phase. The hydrophilic–lipophilic balance (HLB) is an important basis for screening surfactants [47,48]. When HLB value is between 8 and 18, it can be used as emulsifier for O/W microemulsion, which is of our interest. Thus, we mixed two surfactants, Tween80 (HLB =1 5.0) and Span80 (HLB = 4.3), and evaluated their performance in different ratios, including 4:6, 5:5, 6:4, 7:3, 8:2, 9:1, and 10:0 (shown in Table 1). The oil solvent was set as isopropyl myristate (IPM) at first (other solvents were explored later), the ratio of krill oil and solvent was fixed as 1:3, the co-surfactant was ethanol, and the mass ratios of surfactant to co-surfactant (Km) value was 3:1. For each ratio of surfactants, 10 systems with the surfactant phase to oil phase ratio of 9:1-1:9 were prepared, the critical point of forming O/W microemulsion was determined for each system, and pseudo-ternary phase diagrams were drawn. The ratio of two surfactants with the largest microemulsion area in the diagram was chosen. Figure 1 and Figure 2 show percentages of microemulsion area in the total phase diagram for each ratio of surfactants.

**Figure 3:**
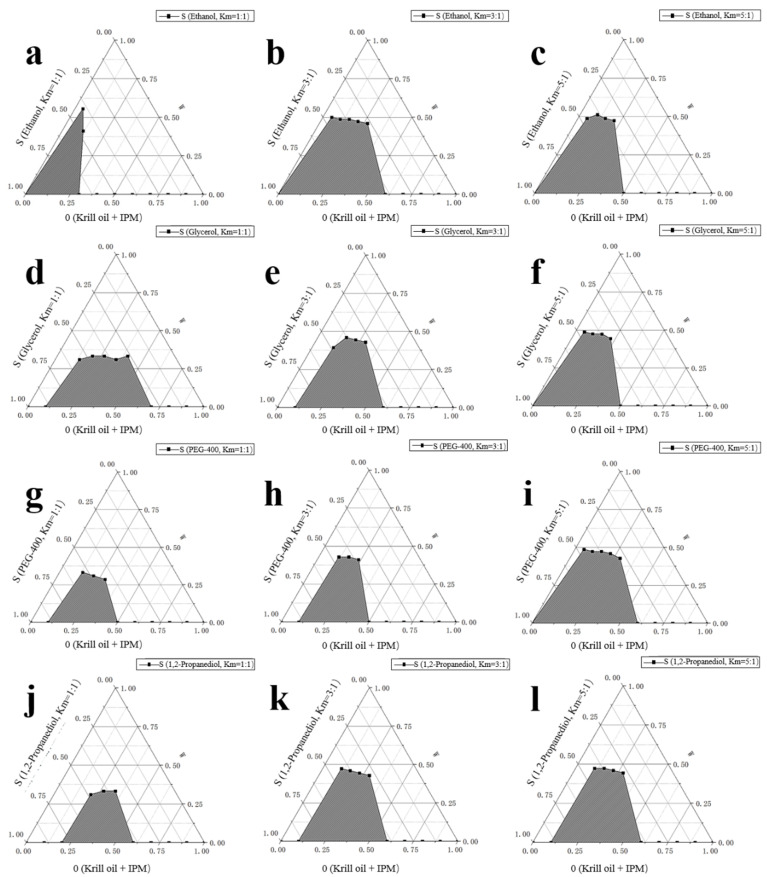
Microemulsion area in phase diagram for each combination of co-surfactant and Km value (mass ratio of surfactant to co-surfactant). (**a**–**c**) Co-surfactant: Ethanol with Km value 1:1, 3:1, and 5:1, respectively. (**d**–**f**) Co-surfactant: Glycerol with Km value 1:1, 3:1, and 5:1, respectively. (**g**–**i**) Co-surfactant: macrogol 400 (PEG-400) with Km value 1:1, 3:1, and 5:1, respectively. (**j**–**l**). Co-surfactant: 1,2-propanediol with Km value 1:1, 3:1, and 5:1, respectively. S refers to the content of surfactants (including surfactant and co-surfactant) in the system; O refers to the content of oil phase [including krill oil (KO) and isopropyl myristate (IPM)] in the system; and W refers to the content of water in the system. Km value refers to the mass ratio of surfactant to co-surfactant. All microemulsions were formed in 25 ± 1 °C, unless otherwise specified.

**Figure 4:**
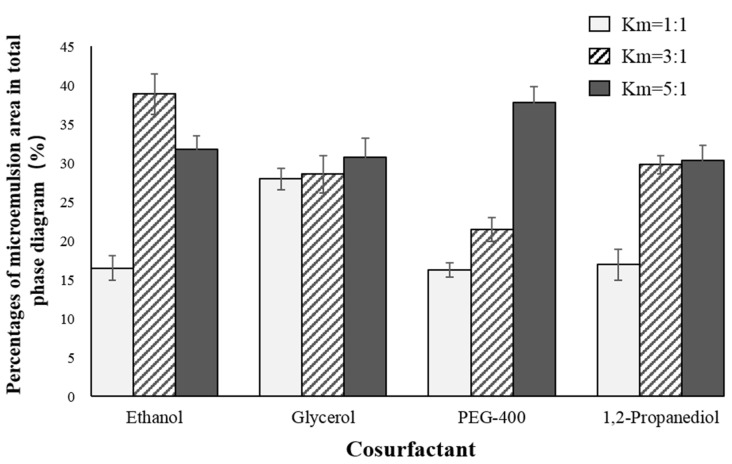
Percentages of microemulsion area in total phase diagram for each combination of co-surfactant and Km value. Km value refers to the mass ratio of surfactant to co-surfactant.

According to Figure 2, we chose Tween80:Span80 = 8:2 as the ratio of two surfactants and we then continued to explore the effects of four co-surfactants [ethanol, glycerol, 1,2-propanediol, macrogol 400 (PEG-400)] and three Km values (1:1, 3:1, 5:1) on the formation of microemulsions. Similarly, we fixed the solvent as IPM, the ratio of krill oil and solvent as 1:3, and Tween80:Span80 as 8:2. For each selected co-surfactant and Km value, 10 systems with a ratio of surfactant phase to oil phase of 9:1–1:9 were prepared and the critical points for the formation of O/W microemulsion were determined. Figure 3 and Figure 4 present percentages of microemulsion area in the total phase diagram for each combination of co-surfactant and Km value.

Overall, ethanol had the best performance, especially under the Km values of 3:1 and 5:1. Considering ethanol is also more commonly used when compared to other co-surfactants, we finally chose ethanol as the co-surfactant, with Km value of 3:1.

**Figure 5:**
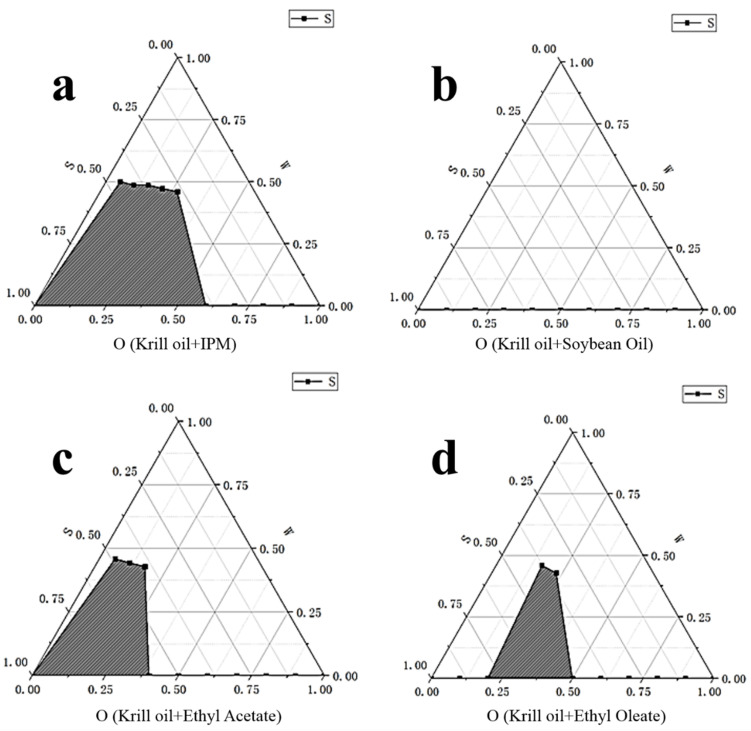
Percentages of microemulsion area in total phase diagram for each oil solvent. (**a**). Solvent: Krill oil + IPM. (**b**) Solvent: Krill oil + soybean oil. (**c**) Solvent: Krill oil + ethyl acetate. (**d**) Solvent: Krill oil + ethyl oleate. S refers to the content of surfactants in the system; O refers to the content of oil phase in the system; and W refers to the content of water in the system. There was no microemulsion formed in (**b**). All microemulsions were formed in 25 ± 1 °C, unless otherwise specified.

Due to the large molecular weight and poor fluidity of Antarctic krill oil, it needs a suitable solvent to dissolve it first. Based on solubility and safety considerations, isopropyl myristate (IPM), ethyl acetate, soybean oil, and ethyl oleate were preliminarily selected as the preselected oil phase solvents for research. Still, the ratio of krill oil and solvent was fixed as 1:3, Tween80:Span80 was fixed as 8:2, and ethanol was chosen as co-surfactant, with Km value of 3:1, as determined previously. For each selected solvent, 10 systems with a ratio of surfactant phase to oil phase of 9:1–1:9 were prepared, and the critical points for the formation of O/W microemulsion were determined. Figure 5 presents percentages of microemulsion area in total phase diagram for each solvent (mentioned in the legend of the figure).

For our final formula of krill oil O/W microemulsion, we chose IPM as solvent, and the ratio of krill oil and solvent was 1:3. Tween80 and Span80 were mixed at a ratio of 8:2 and ethanol was chosen as the co-surfactant with Km value of 3:1. The pseudo-ternary phase diagram of the best formula is presented in Figure 1e.

### 2.2. Characteristics of Prepared Antarctic Krill Oil O/W Microemulsion

#### 2.2.1. Electrical Conductivity

**Figure 6:**
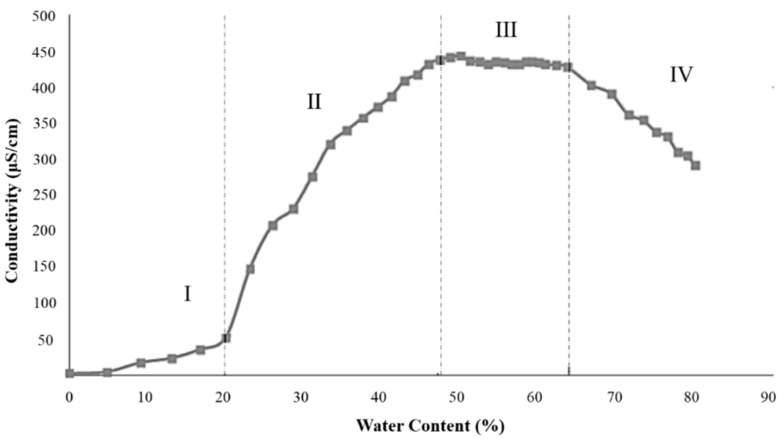
Electrical conductivity of the microemulsion with the increase of water content. Region I: Water content of 0% to 20%, the system was W/O microemulsion. Region II: Water content of 20% to 47%, the system shifted from the W/O structure to bicontinuous structure. Region III: Water content of 47% to 64%, the system began to convert into the O/W microemulsion. Region IV: Water content of 64% to 80%, the system was O/W microemulsion.

For the best formula, we explored the change of electrical conductivity when water content of the microemulsion area increased from 0% to 80%. The change of electrical conductivity can better characterize how the structure of microemulsion transformed from water-in-oil (W/O) to bicontinuous (BC) and then to oil-in-water (O/W). Figure 6 represents change of electrical conductivity with the increase of water content, consistent with the electrical conductivity result of food-grade vitamin E microemulsions reported by Feng [49].

In region I, corresponding to the water content of 0% to 20%, the electrical conductivity increased slowly and almost linearly. The system was W/O microemulsion and the slow increase of electrical conductivity may have been due to the hydration of surfactant hydrophilic head by water molecules. There was no free water in continuous phase and very limited conductive droplets. In region II, corresponding to the water content of 20% to 47%, the conductivity increased rapidly, which was due to the gradual shift from the W/O structure to bicontinuous structure. In this state, the number of conductive droplets increased and the space among droplets decreased. The interaction of conductive droplets formed the conductive chain. In region III, corresponding to the water content of 47% to 64%, the electrical conductivity of the microemulsion system reached the maximum value. The system gradually began to convert into the O/W microemulsion, and water became the continuous phase of the system instead of local continuity of the oil phase and the water phase. In region IV, when the water content was between 64% and 80%, the system was O/W microemulsion. With the increase of water content, the electrical conductivity gradually decreased because of dilution.

#### 2.2.2. Transmission Electron Microscopy (TEM) and Cryogenic TEM (Cryo-TEM)

Traditional TEM is a commonly used method for observing the microscopic morphology of samples, but it also has many limitations. For example, dyeing is required, the morphology of liquid samples will be affected to a certain extent when observing, due to vacuum state at room temperature; and electron beam damage to the sample also exists.

Cryo-TEM has become a more advantageous tool for evaluating the structure and morphology of nanoscale samples compared to traditional TEM. The advantage of cryo-TEM over other related techniques is that it enables sample visualization in native conditions at a lower temperature without staining, makes water directly into vitrification ice, and reduces the damage of the electron beam to the sample so that it keeps the original shape of the sample as much as possible, especially for a liquid sample [50,51].

**Figure 7:**
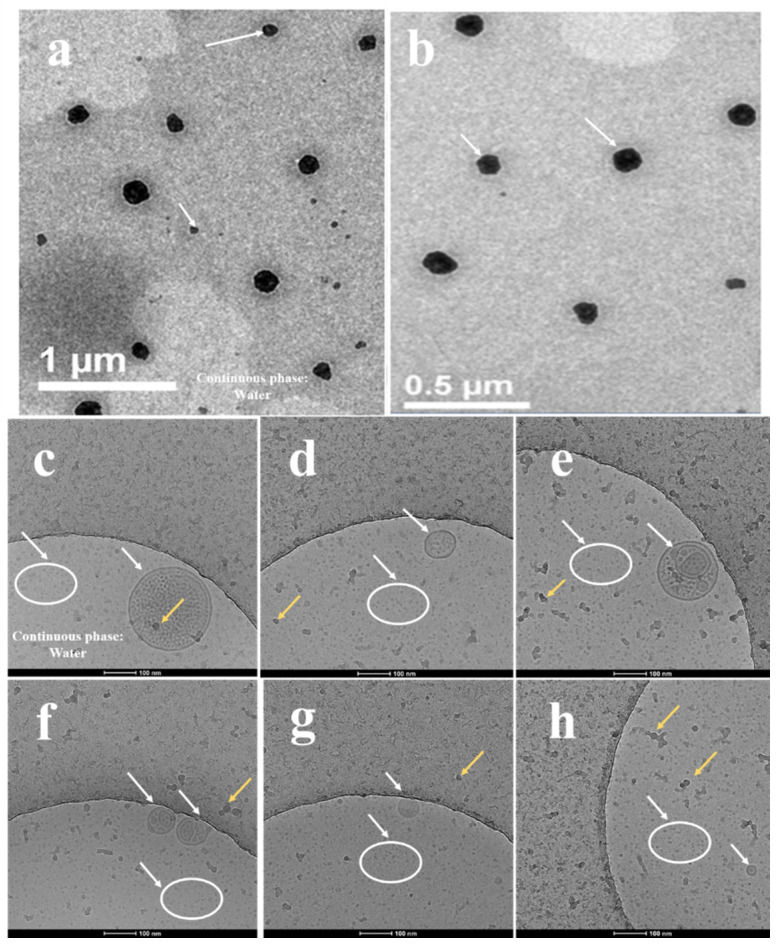
Microstructure obtained by transmission electron microscope (TEM) and cryogenic TEM (Cryo-TEM) with water content of 60%. (**a**,**b**) TEM images of KO microemulsion. The scale bars are 1 μm and 0.5 μm, respectively. (**c**–**h**) Cryo-TEM images of KO microemulsion. The scale bars are 100 nm. White arrows (**a**–**h**) and white circles (**c**–**h**) point toward droplets of microemulsion. Yellow arrows (**c**–**h**) point toward ice crystals deposited on the grid and samples after vitrification.

Figure 7a,b is the result of TEM. The result shows that droplets of microemulsion were almost spherical with droplet sizes of less than 100 nm. The specific droplet sizes need to be combined with the measurement results of the size analyzer in the next section. As for the morphology, it has been reported that there is a difference between microemulsion and ordinary nanoemulsion in microscopic morphology, which mainly manifested in the fact that microemulsion droplets may present a nonspherical shape due to lower interfacial tension, while nanoemulsion droplets usually present a spherical or oval shape affected by the Laplace force. The distribution of droplets was uniform. The TEM result was consistent with the results of microstructure of emulsion reported by Jee Hye Kim [52], and again confirmed the formation of microemulsion. Compared to traditional TEM, the edges of the droplets were smoother, observed by cryo-TEM (Figure 7c–h). Interestingly, we found several internal morphologies of droplets that were not observed under TEM (Figure 7c–f). As far as we know, in an oil-in-water microemulsion system, the lipophilic tail group of the surfactant aggregates to form a hydrophobic core, while the hydrophilic head group was in contact with water phase on the outside, and oil molecules could be distributed between the tails of the surfactant or directly enter the hydrophobic core. The latter was exactly the situation we could observe in cryo-TEM images. As reported by Roger [53], as water diffuses in, the morphology of each droplet underwent a series of transformations prepared by the phase inversion composition emulsification method. At first, oil droplets were wrapped by water phase, as shown in Figure 7c,d. The droplets were usually larger than those of the following stages. With further water swelling, reverse micelles were formed, the curvature decreased, and the lamellar phase began to form. It could be observed that the lamellar phase and the first stage coexist, in Figure 7e,f. As the curvature turned toward the oil, the lamellar phases were connected to form a sponge phase, the microemulsion droplets nucleated in the sponge phase, and their diameter matched the curvature. In the final stage, the rest of the sponge phase became a collection of very small micelles, large droplets were released, and droplets with normal small sizes were formed, as shown in Figure 7h and white circles marked in Figure 7c–h. Although we did not capture all the stages of the transformation of droplets, our observations were basically consistent with Roger’s report and explicitly displayed the formation process and different stages of microemulsion droplets, indicating that cryo-TEM did provide us with new ideas and new ways to explore the transformation and clearer morphology of microemulsion droplets we prepared.

#### 2.2.3. Droplet Size Analysis

**Figure 8:**
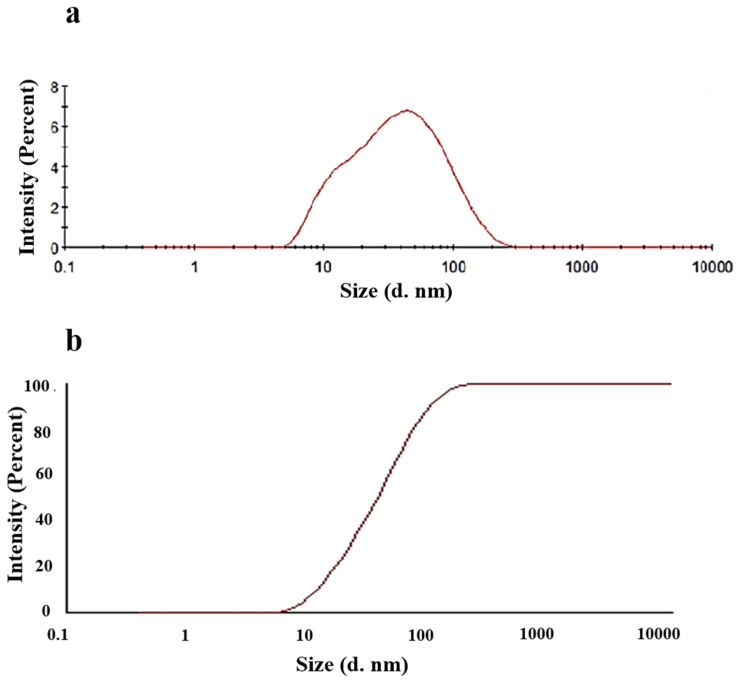
Size distribution and its integral curve of prepared krill oil oil-in-water(O/W) microemulsion by intensity. (**a**). Size distribution. (**b**) Integral curve of the size distribution.

We characterized the distribution of droplet size in the microemulsion. Figure 8a shows the size distribution by intensity. The distribution of the droplet size presented a single peak at 47.52 nm. The z-average of the system, that is, the average particle size, was 27.47 nm, and the polydisperse index (PDI) was 0.381. Figure 8b, which is the integral curve of droplet size, also shows that more than 90% of the droplet of the microemulsion was less than 100 nm. We also presented the size distribution by number (Figure S1) and size distribution by volume (Figure S2) in Supplementary Materials.

#### 2.2.4. Rheological Characteristics

We also explored rheological characteristics of prepared O/W krill oil microemulsion. The viscosity of four samples with water contents of 50%, 60%, 70%, and 80%, respectively, was tested with change of shear rate. The range of shear rate was between 10^−2^s^−1^–10^1^s^−1^ and the temperature was fixed at 25 °C.

**Figure 9:**
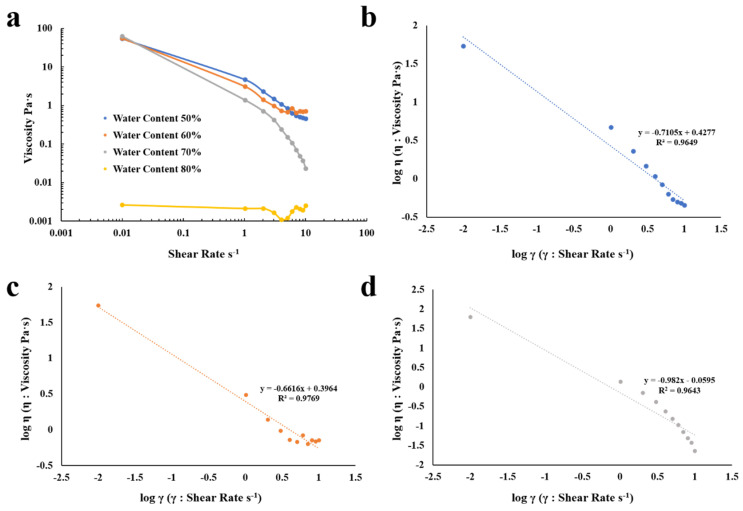
The viscosity of microemulsion at different shear rates and their fitted curves with 50%, 60%, 70%, and 80% water content. (**a**) Viscosity-shear rate profiles of microemulsions with water content of 50%, 60%, 70%, and 80%. (**b**) Fitted curves of 50% water content microemulsion. (**c**) Fitted curves of 60% water content microemulsion. (**d**) Fitted curves of 70% water content microemulsion.

Figure 9a shows the changes of viscosity with shear rates. Samples with water content of 50%, 60%, and 70% showed a steep decline at a low shear rate (10^−2^s^−1^–10s^−1^) and, with the increase of the water content, the viscosity decreased more significantly at a low shear rate. At a higher shear rate (3s^−1^–10s^−1^), the viscosity of samples with water content of 50%, 60%, and 70% remained basically constant. However, the viscosity of sample with 80% water content remained almost unchanged in the whole shear rate range. So, we fit to the experimental curves of microemulsions with water content of 50%, 60%, and 70%, shown in Figure 9b–d.

In order to further understand the rheological properties of the microemulsion, the Ostwald-De Waele power law was used to fit the viscosity of samples with water content of 50%, 60%, and 70%.

Ostwald-De Waele power law [54] can be presented as:

η = τ/γ = (kγ^n)/γ = kγ^(n − 1)

where η represents the viscosity, τ represents the shear force, k represents the flow consistency index, γ represents the shear rate, and n represents the flow behavior index.

By taking the logarithm of both sides of the relationship, we can then choose log η as y-axis and log γ as x-axis for linear fitting.

logη = logk + (n − 1)logγ

The flow behavior index (n) of samples with water content of 50%, 60%, and 70% was 0.0351, 0.3384, and 0.018, respectively. Since the value of n is smaller than 1, samples are pseudoplastic fluid and have shear thinning property, which is beneficial for further application of food and drug dispersions. For example, reduced viscosity facilitates administration and microemulsion preparation. At a high shear rate, the highly entangled system can be transformed into a more ordered system and the forces between droplets can be reduced. This has potential impact on the release of active components of the system.

#### 2.2.5. Thermal Behavior

**Figure 10:**
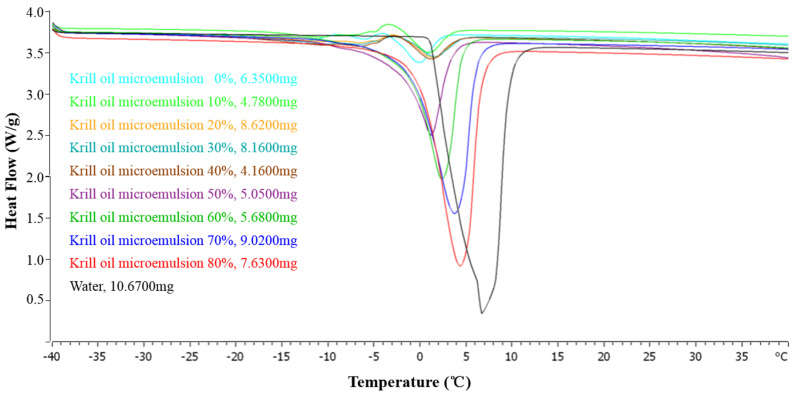
Differential scanning calorimetry (DSC) thermogram of microemulsions with different water contents (0%, 10%, 20%, 30%, 40%, 50%, 60%, 70%, and 80%) and ultrapure water. (The peak upwards is exothermic and downwards is endothermic.).

Krill oil microemulsions with water content of 50%, 60%, 70%, and 80% are oil-in-water microemulsions, while water content of 0–40% are water-in-oil type and bicontinuous type. Their differential scanning calorimetry (DSC) thermograms with ultrapure water as reference are shown in Figure 10.

When the water contents were below 40%, the DSC curves of the samples had less obvious endothermic or exothermic peaks. At this time, the water in the system existed as combined water, a small amount of bound water, bound water, and interface water connected by the hydrophilic group of the surfactants. The oil served as the continuous phase and the water was wrapped in the internal phase so that the interaction between water and surfactants was strong [55].

It was found easily that the DSC spectrum of the microemulsion system with lower water contents (0–40%) had extremely small exothermic or endothermic peaks, indicating that the water in the system belonged to the "nonfrozen water" with very low activity, usually in the water core, and was confined in it. It is worth noting that as the water content increased, the onset temperature of the exothermic peak increased. This may be because as the water content increased, the amount of nonfrozen water directly bound to the hydrophilic group of Tween80 increased. The mobility of the molecules decreased, resulting in a higher temperature required to rearrange the emulsifier molecules. In addition, the enthalpy values of the exothermic peak gradually decreased. On the one hand, it may have been caused by the dilution effect. On the other hand, as the content of nonfrozen water bound to the hydrophilic group of the emulsifier increased, the emulsifier could not be rearranged normally [56]. As a result, the peak value dropped. Therefore, the additional exothermic peak proposed was more likely to be attributed to the surfactant phase and bounded water of microemulsion system and complied with water-in-oil characteristics.

As the water content increased, the water droplets in the dispersed phase increased and the free water increased. Only one endothermic peak was shown between −40 °C to 40 °C for all O/W microemulsions and ultrapure water. When the water content was greater than 50%, the obvious endothermic peak appeared near 0~10 °C, which was the melting temperature of free water in the microemulsion. As the water content increased, the peak starting temperature shifted to the right and the endothermic peak area gradually increased. Their diagrams were closer to that of ultrapure water, indicating that the free water content in the microemulsion gradually increased [57] and the system gradually transformed into O/W microemulsion as the amount of water increased.

**Figure 11:**
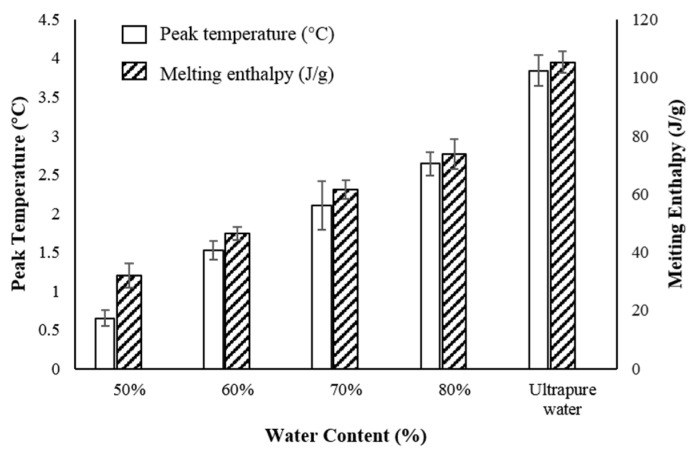
Endothermic peaks for O/W microemulsions with different water contents (50%, 60%, 70%, and 80%) and ultrapure water.

This result indicated that, although there was combination between some water molecules and the interfacial layer of the dispersed phase, the force between them indeed decreased obviously, so that the content of free water in the system further increased. Further analysis of endothermic peaks of each sample is summarized in Figure 11.

### 2.3. Stability of Prepared Antarctic Krill Oil O/W Microemulsion

#### 2.3.1. Effect of pH on the Stability of Krill Oil Microemulsion

**Figure 12:**
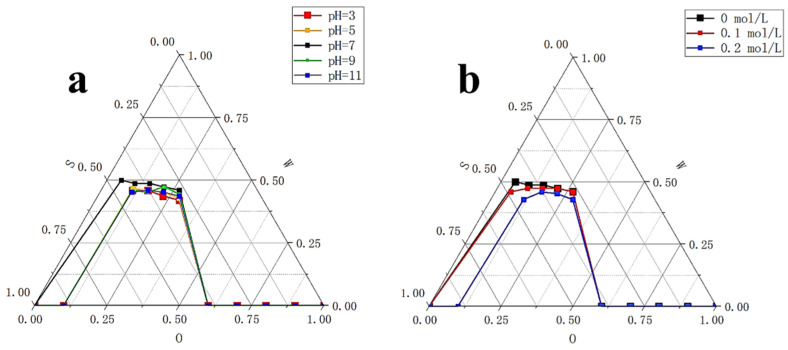
Pseudo-ternary phase diagram of krill oil microemulsion under different pH and NaCl concentration. (**a**) Pseudo-ternary phase diagram of krill oil microemulsion under different pH (3, 5, 7, 9, and 11). (**b**) Pseudo-ternary phase diagram of krill oil microemulsion under different NaCl concentration. S refers to the content of surfactants in the system; O refers to the content of oil phase in the system; and W refers to the content of water in the system. All microemulsions were formed in 25 ± 1 °C, unless otherwise specified.

According to Figure 12a, when compared to neutral condition, microemulsion area in pseudo-ternary phase diagram became smaller under acidic or alkaline conditions. This was mainly due to failure of the system with a ratio of surfactant phase to oil phase of 9:1 to form microemulsion under acidic or alkaline conditions. Because the microemulsion was most stable under neutral condition, under other pH condition the area under curve was slightly reduced, (mainly affecting the position of the leftmost point in the pseudo-ternary phase diagram, resulting in a different diagram with pH 7) and systems with other surfactant phase to oil phase ratios were not impacted much. However, the amount of reduction was small, which could support stability of microemulsion under different pH conditions. When pH dropped from 5 to 3 or increased from 9 to 11, microemulsion area in pseudo-ternary phase diagram did not change much. Acidic and alkaline conditions had similar effect on our microemulsion. Overall, the O/W krill oil microemulsion had a certain level of tolerance to pH changes.

#### 2.3.2. Effect of Salinity on the Stability of Krill Oil Microemulsion

The reason why it was necessary to explore the stability of microemulsions under different salinities is mainly because the content of inorganic salts in surfactant microemulsions is known to greatly affect the phase behavior [49]. Since food comes into contact with a large amount of salt when it enters the human body, we should understand the behavior of microemulsions under the influence of salinity. From Figure 12b, when NaCl solution concentration was 0.1mol/L, the microemulsion area in the pseudo-ternary phase diagram was basically the same as that with the ultrapure water. However, when NaCl solution concentration increased to 0.2 mol/L, the microemulsion area in the pseudo-ternary phase diagram decreased. This was also because the system with a ratio of surfactant phase to oil phase of 9:1 failed to form microemulsion under this condition. Other systems were not impacted much, consistent with the research of influence of salinity on the microemulsion system [49]. Overall, our O/W krill oil microemulsion had good salt tolerance, indicating that the property of KO microemulsion would not be changed due to the contact between surfactant and inorganic salt when it entered the human body.

#### 2.3.3. Effects of Storage Time and Temperature on the Stability of Krill Oil Microemulsion

**Figure 13:**
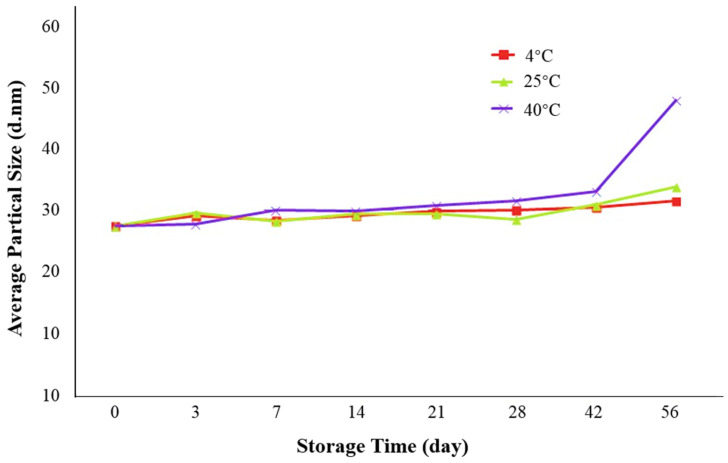
Average droplet size of krill oil microemulsion under different storage time and temperature.

From Figure 13, between day 0 and day 28, no obvious changes in average droplet size were seen in all samples stored under 4 °C, 25 °C, or 40 °C and the three groups remained similar. After day 28, samples under 4 °C and 25 °C were still quite stable and average droplet size did not exceed 35 nm for the whole storage period. The average increase of droplet size was 14.60% and 22.82% for samples under 4° C and 25 °C, respectively. Samples stored under 40 °C had a relatively obvious increase in average droplet size, especially from day 42 to day 56. However, the average droplet size still did not exceed 50 nm at the end of the storage period. Therefore, our O/W krill oil microemulsion retained high stability under refrigeration and room temperature and even under relatively high temperature its overall stability was not destroyed.

## 3. Materials and Methods

### 3.1. Materials

Antarctic krill oil from Antarctic krill (Euphausia superba) was supplied by Keruier Biological Technology Co., Ltd. (Jinan, China), stored at 2–5 °C throughout the experiment.

Tween80 (Polyoxyethylene Sorbitan Monooleate, pharmaceutical grade), Span80 (Sorbitan Monostearate, pharmaceutical grade), IPM (isopropyl myristate, 98%), Ethanol (pharmaceutical grade, 99.5%), 1,2-propanediol [analytial reagent (AR), 99%], glycerol [American Chemical Society (ACS), ≥99.5%], soybean oil (pharmaceutical grade), ethyl acetate [guaranteed reagent (GR), 99.5%], ethyl oleate [for gas chromatography (GC) purpose], potassium hydroxide (GR, 95%), sodium hydroxide (AR), and phosphotungstic acid 44-hydrate (99.5%) were purchased from Aladdin Bio-Chem Technology Co., LTD (Shanghai, China). PEG-400 (polyethylene glycol, Bioultra, 400) was purchased from Sigma Chemical Co., Ltd. (St. Louis, MO, USA). Acetic acid (AR) and sodium chloride (AR, ≥99.5%) were purchased from Hushi Laboratorial Equipment Co., Ltd. (Shanghai, China). All components were used without further purification. The water was ultrapure and was supplied by Suitian Enviro-Tech Co., Ltd. (Shanghai, China).

### 3.2. Methods

#### 3.2.1. Formula Selection of the Microemulsions

Shah method [58] is a conventional preparation method of microemulsion, which mixes oil phase, surfactant, and co-surfactant into one emulsifying system and adds water to the system gradually. The speed of agitation was set at 800 r/min. When water is gradually added to the system, the viscosity increases first and then suddenly decreases, forming clear O/W microemulsion of Antarctic krill oil [44,59,60].

**Figure 14:**
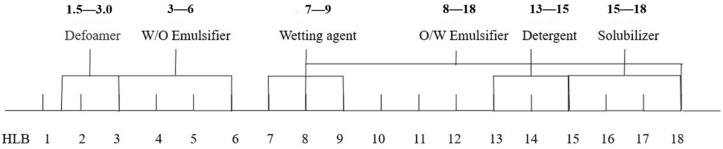
The usage of different HLB values.

There were three parts of experiments to select the best formula of krill oil microemulsion, including selection of surfactant [42,61,62], co-surfactant, and oil phase. Considering Span80 is a lipophilic emulsifier with an HLB of 4.3 and Tween80 is a hydrophilic emulsifier with an HLB of 15.0, the HLB range of compounded surfactants was larger by using Tween80 and Span80 together, which could take both the affinity for the oil phase and the water phase into account, and was more conducive to the formation and stability of the microemulsion (Figure 14). Two surfactants, Tween80 and Span80, were mixed in different ratios, including 4:6, 5:5, 6:4, 7:3, 8:2, 9:1, and 10:0. By comparing the size of microemulsion area in pseudo-ternary phase diagrams [63], the optimal ratio of two surfactants was determined. Then four kinds of co-surfactants (ethanol, glycerol, 1,2-propanediol, PEG-400) and three mass ratios of surfactant to co-surfactant (1:1, 3:1, 5:1) were chosen to select the best kind of co-surfactant and its dosage by pseudo-ternary phase diagrams. Finally, four kinds of solvents (IPM, soybean oil, ethyl acetate, ethyl oleate) were screened also by pseudo-ternary phase diagrams to solubilize krill oil, and the ratio of krill oil and solvent was fixed as 1:3. For every formula requiring selection, 10 mixtures, in which ratio of surfactant phase (surfactant and co-surfactant) to oil phase (krill oil and solvent) varied from 9:1 to 1:9 were prepared, respectively. The total volume of prepared microemulsions was 50 mL every time (amounts of reagents are shown in Table 2). Every mixture was stirred at 25 ± 1 °C at 800 r/min using a digital thermostatic magnetic stirrer from Yuexin instrument manufacturing Co., Ltd. (Changzhou, China) and ultrapure water was added slowly to prepare krill oil microemulsions. We recorded the points when O/W microemulsions formed and drew the pseudo-ternary phase diagrams using Origin (OriginLab Corp., Northampton, MA, USA). Microemulsion areas in pseudo-ternary phase diagrams were calculated using Image-Pro Plus 6.0 (Media Cybernetics Inc., Bourne End, UK) and the formula with the largest microemulsion area was chosen for further exploration.

#### 3.2.2. Electrical Conductivity

We prepared krill oil micromemulsion with a formula determined in Section 2.2 to establish the relationship between electrical conductivity (µS/cm) and water content (0–80%). The Shah method was still used, and temperature was controlled at 25 ± 1 °C. Every time when 1 mL ultrapure water was added to the system, we continued to stir for 5 min and measured electrical conductivity using a conductivity meter (CT3030, Kedida, Shenzhen, China).

#### 3.2.3. Transmission Electron Microscopy (TEM) and Cryo-TEM

To explore the microstructure of prepared krill oil O/W microemulsion, we used JEM-2100 transmission electron microscope (JEOL Ltd., Japan) and Talos F200c cryogenic transmission electron microscope (cryo-TEM) (FEI Company, Fremont, CA, USA).

A copper mesh with support film was placed on the stencil and 2 µL of the diluted microemulsion to be tested was added and dried naturally. Then, 2% phosphotungstic acid solution with pH adjusted to 7.4 was prepared and added on the stencil. We put the dried copper mesh into the dye solution for negative dyeing for 15 min, absorbed the excess dye solution using filter paper, then rinsed with ultrapure water, and dried using filter paper. We repeated the process for 2–3 times and dried the sample statically. The morphology of krill oil microemulsion was observed under TEM.

We diluted the sample to 10 mg/mL and prepared the sample for cryo-TEM observation in a vitrification system with strict environmental control (Vitrobot, FEI). Before applying the sample, the 300-mesh carbon-coated copper grid was subjected to glow discharge treatment for 25 s, and then 3 μL of the sample was dripped onto the grid. Blot time was 3.5 s, blot force was 3, wait time was 3 s, and we the sample was immediately frozen in liquid ethane. After preparation, we placed the vitrified sample in liquid nitrogen until it was inserted into the Cryo-TEM holder. During visualization, we kept the sample temperature at −178 °C and observed with Talos F200c running at 200 kV (FEI), using Ceta 4k × 4k multiscan to take images.

#### 3.2.4. Droplet Size Analysis

To characterize the droplet size of O/W microemulsion, the prepared microemulsion was diluted 10 times with ultrapure water and homogenized with JY92-II DN ultrasonic cell pulverizer (Xinzhi biotechnology co., LTD, Ningbo, China) for 30 min. Considering that the microemulsion droplets we prepared were all nanometers-level, Zetasizer Nano ZS90 (Malvern Panalytical, Malvern, UK) was used by dynamic light scattering for droplet size analysis.

#### 3.2.5. Rheological Characteristics

We used MCR302 rotary rheometer (Anton Paar GmbH, Graz, Austria) with cone plate system to explore how the viscosity of selected krill oil microemulsion changed with different shear rates under different water content. Temperature was controlled at under 25 °C. We were only interested in rheological characteristics of O/W microemulsion. The selected formula transformed into O/W type when water content was around 47%. Thus, microemulsions with water content of 50%, 60%, 70%, and 80% were prepared as samples.

#### 3.2.6. Thermal Behavior

We adopted the DSC1 differential scanning calorimeter (METTLER TOLEDO, USA) to explore thermal behavior of krill oil microemulsion. For each sample, about 5–10 mg were put into a standard aluminum crucible for weighing, then covered with perforated sample, and sealed with a lid press. The sealed crucible was placed into the sample tray of the calorimeter, and the pressed empty dish was placed on the reference side. Heating rate was set at 10 °C/min with temperature range from −40 °C to 40 °C.

#### 3.2.7. Stability

Three aspects of stability were evaluated, including pH stability, salinity stability, and storage stability. For pH stability, acid buffer solutions of pH = 3 and pH = 5 were prepared with acetic acid, alkaline buffer solutions of pH = 9 and pH = 11 were prepared with potassium hydroxide, and ultrapure water of pH = 7 was used as the neutral control. Microemulsions with selected formula were prepared as described in Section 2.2, except for using different buffer solutions instead of ultrapure water. Microemulsion areas in pseudo-ternary phase diagrams were adopted to compare stability under different pH values [64].

Similarly, to explore salinity stability, NaCl solutions of 0.1 mol/L and 0.2 mol/L were prepared, respectively, and ultrapure water was still used as the control [49]. We prepared microemulsions as described in Section 2.2 and substituted ultrapure water with different NaCl solutions. Microemulsion areas in pseudo-ternary phase diagrams were adopted to compare stability under different concentrations of NaCl.

We evaluated the influence of temperature and time on storage stability. Microemulsions were prepared as described in Section 2.2 and three samples were stored in a refrigerator at 4 °C, under room temperature (25 °C) and in water bath (40 °C), respectively. On days 0, 3, 7, 14, 21, 28, 42, and 56, Zetasizer Nano ZS90 (Malvern Panalytical, UK) was used for droplet size analysis, and average droplet size was used as the main index to characterize the influence of storage time and temperature.

## 4. Conclusions

In this study, we used the Shah method to prepare the oil-in-water microemulsion of Antarctic krill oil. The best formula selected by comparing the size of the microemulsion area in the pseudo-ternary phase diagram was: Antarctic krill oil:IPM = 1:3 as the oil phase, Tween 80:Span 80 = 8:2 as surfactant, ethanol as co-surfactant, and the mass ratio of surfactant to co-surfactant (Km) was 3:1. A series of characteristics were characterized. The electrical conductivity changed with water content, showing that the microemulsion converted from a W/O type to a bicontinuous type and finally converted to an O/W type. The electrical conductivity increased slowly at first, then increased rapidly, and, after reaching the maximum, it remained basically stable and finally decreased. By TEM and droplet size analysis, we observed that the droplets were mostly spherical, the dispersion was uniform, and the sizes of droplets were mostly less than 100 nm. Rheological characteristics were assessed using a rotary rheometer, which found that the prepared microemulsion was a pseudoplastic fluid with shear thinning characteristics when the water content was 50%, 60%, and 70%. This property is advantageous for dispersion system in food or medicine. The DSC spectrum showed that the free water content in O/W microemulsion increased largely and the diagram was close to that of ultrapure water. The stability evaluation experiment showed that the prepared Antarctic krill oil microemulsion had certain salt, acid, and alkali tolerance, and there was no significant change in the quality of the microemulsions under refrigeration, room temperature, or 40 °C for one month. It was seen that the O/W Antarctic krill oil microemulsion prepared had a series of ideal characteristics that provide a basis for future application.

## Figures and Tables

**Table 1:** Hydrophilic-lipophilic balance (HLB) value at different ratios of Tween 80 to Span 80.

Tween 80:Span 80	4:6	5:5	6:4	7:3	8:2	9:1	10:0
HLB value	8.58	9.65	10.72	11.79	12.86	13.93	15.00

**Table 2:** Amouts of reagents for different formula.

**Example 1. Ratio of Surfactant Phase (Surfactant and Cosurfactant) to Oil Phase (Krill Oil and Solvent): 8:2**						
		Tween 80/g	Span 80/g	Oil solvent/g	Krill oil/g	Cosurfactant/g
Mass ratios of Tween 80 to Span 80	4:6	4.8	7.2	3.0	1.0	4.0
	5:5	6.0	6.0			
	6:4	7.2	4.8			
	7:3	8.4	3.6			
	8:2	9.6	2.4			
	9:1	10.8	1.2			
	10:0	12.0	0.0			
Mass ratios of surfactant to cosurfactant	1:1	9.6	2.4	4.5	1.5	12.0
	3:1			3.0	1.0	4.0
	5:1			2.7	0.9	2.4
**Example 2. Ratio of Surfactant Phase (Surfactant and Cosurfactant) to Oil Phase (Krill Oil and Solvent): 5:5**						
		Tween 80/g	Span 80/g	Oil solvent/g	Krill oil/g	Cosurfactant/g
Mass ratios of Tween 80 to Span 80	4:6	4.8	7.2	12.0	4.0	4.0
	5:5	6.0	6.0			
	6:4	7.2	4.8			
	7:3	8.4	3.6			
	8:2	9.6	2.4			
	9:1	10.8	1.2			
	10:0	12.0	0.0			
Mass ratios of surfactant to cosurfactant	1:1	9.6	2.4	18.0	6.0	12.0
	3:1			12.0	4.0	4.0
	5:1			10.8	3.6	2.4

Note: Only use two ratios (8:2 and 5:5) for calculation display, because for every formula requiring selection, all mixtures, in which ratio of surfactant phase (surfactant and co-surfactant) to oil phase (krill oil and solvent) varied from 9:1 to 1:9 were needed. The total volume was 50 mL every time (for example, water content 60%, took 20 mL mixtures, displayed in table, and added 30 mL water in the system gradually). Mass ratios of Tween 80 to Span 80 was 8:2 and mass ratios of surfactant to co-surfactant was 3:1, unless otherwise specified.

## Supplementary Materials

The following are available online at https://www.mdpi.com/1660-3397/18/10/492/s1, Figure S1: Size distribution of prepared krill oil O/W microemulsion by number; Figure S2. Size distribution of prepared krill oil O/W microemulsion by volume.
