# Supplementary material for: Preparation and Characterization of Microemulsions Based on Antarctic Krill Oil
Source: Mar Drugs. 2020 Sep 25;18(10):492. doi: 10.3390/md18100492 (PMC7601059; doi:10.3390/md18100492)
Supplement: Supplementary file 1 [file marinedrugs-18-00492-s001.pdf]

Supplementary Figures

			Size (d.nm):	% Number:	St Dev (d.nm):
Z-Average (d.nm):	27.47	Peak 1:	7.760	100.0	2.339
PdI:	0.381	Peak 2:	0.000	0.0	0.000
Intercept:	0.929	Peak 3:	0.000	0.0	0.000

Result quality : Good

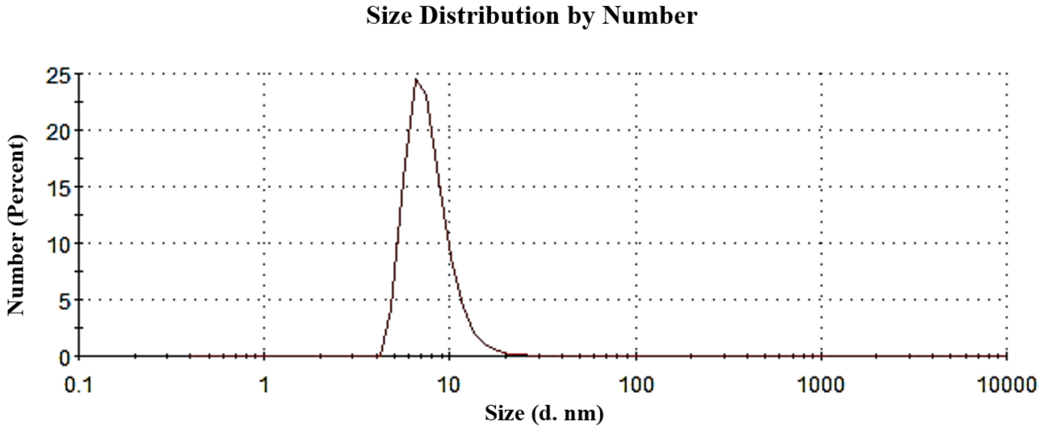

Figure S1. Size distribution of prepared krill oil O/W microemulsion by number

Z-Average (d.nm):	27.47
PdI:	0.381
Intercept:	0.929
Result quality :	Good

	Size (d.nm):	% Volume:	St Dev (d.nm):
Peak 1:	11.28	100.0	7.206
Peak 2:	0.000	0.0	0.000
Peak 3:	0.000	0.0	0.000

Size Distribution by Volume

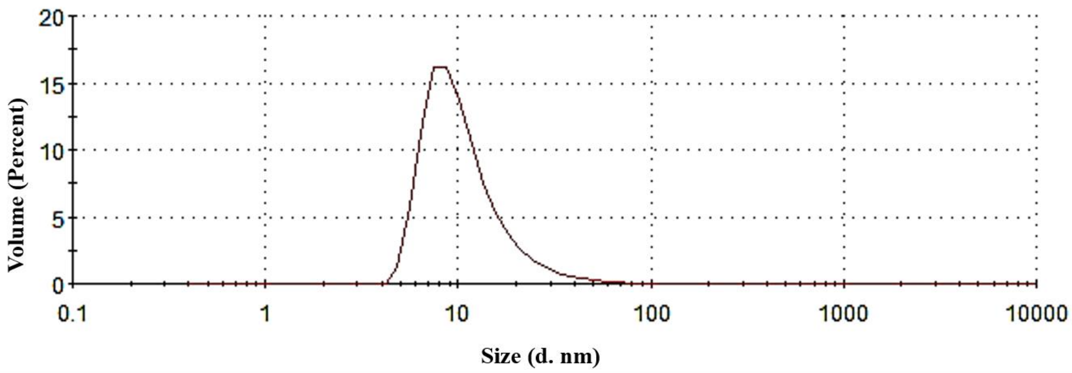

Figure S2. Size distribution of prepared krill oil O/W microemulsion by volume
